# The Composition of Microbiome in Larynx and the Throat Biodiversity between Laryngeal Squamous Cell Carcinoma Patients and Control Population

**DOI:** 10.1371/journal.pone.0066476

**Published:** 2013-06-18

**Authors:** Hong-Li Gong, Yi Shi, Liang Zhou, Chun-Ping Wu, Peng-Yu Cao, Lei Tao, Chen Xu, Dong-Sheng Hou, Yue-Zhu Wang

**Affiliations:** 1 Department of Otolaryngology, Eye, Ear, Nose, and Throat Hospital, Fudan University, Shanghai, China; 2 Department of Clinical Laboratory, Shanghai Pudong Hospital, Fudan University Pudong Medical Center, Shanghai, China; 3 Shanghai Key Laboratory for Reproductive Medicine, Department of Histology and Embryology, Shanghai Jiao Tong University School of Medicine, Shanghai, China; 4 Shanghai-MOST Key Laboratory of Health and Disease Genomics, Chinese National Human Genome Sequencing Centre, Shanghai, China; National Cancer Institute, National Institutes of Health, United States of America

## Abstract

The throat is an ecological assemblage involved human cells and microbiota, and the colonizing bacteria are important factors in balancing this environment. However, this bacterial community profile has thus been poorly investigated. The purpose of this study was to investigate the microbial biology of the larynx and to analyze the throat biodiversity in laryngeal carcinoma patients compared to a control population in a case-control study. Barcoded pyrosequencing analysis of the 16S rRNA gene was used. We collected tissue samples from 29 patients with laryngeal carcinoma and 31 control patients with vocal cord polyps. The findings of high-quality sequence datasets revealed 218 genera from 13 phyla in the laryngeal mucosa. The predominant communities of phyla in the larynx were *Firmicutes* (54%), *Fusobacteria* (17%), *Bacteroidetes* (15%), *Proteobacteria* (11%), and *Actinobacteria* (3%). The leading genera were *Streptococcus* (36%), *Fusobacterium* (15%), *Prevotella* (12%), *Neisseria* (6%), and *Gemella* (4%). The throat bacterial compositions were highly different between laryngeal carcinoma subjects and control population (*p* = 0.006). The abundance of the 26 genera was significantly different between the laryngeal cancer and control groups by metastats analysis (*p*<0.05). Fifteen genera may be associated with laryngeal carcinoma by partial least squares discriminant analysis (*p*<0.001). In summary, this study revealed the microbiota profiles in laryngeal mucosa from tissue specimens. The compositions of bacteria community in throat were different between laryngeal cancer patients and controls, and probably were related with this carcinoma. The disruption of this bio-ecological niche might be a risk factor for laryngeal carcinoma.

## Introduction

The human body is colonized by a large array of microbes, known as human microbiota, that include communities of bacteria, viruses and microbial eukaryotes [1]. Current studies revealing the differences in community composition between individual healthy and unhealthy states are transforming our view of human biology [2], [3]. Each of these microbial communities has their own peculiar profiles, and this ecosystem largely depends on the dynamics of the body microenvironment [4], [5]. Although, this amazingly complex community has enormous impacts on human health it is still poorly understood [6], [7].

The larynx contains numerous bacteria in the respiratory tract. These are known to cause respiratory infections and include the following: *Mycoplasma pneumoniae, Chlamydia pneumoniae, Bordetella pertussis, Haemophilus influenza, and Streptococcus pneumoniae* [8], [9]. The laryngeal mucosa is an ecological niche for various microorganisms [10], and the colonizing bacteria are important factors in maintaining a healthy environment within the human body [11].

To date, the incidence of laryngeal cancer has been relatively stable with approximately 160,000 new cases every year [12]. This type of carcinoma is considerably more frequent in men, and comprises approximately 2.4% of all tumor cases and 2.1% of all tumor deaths worldwide [12]. Laryngeal cancer has a well-proven causal correlation with a smoking and alcohol [12], [13]. Furthermore, it has been suggested that human papillomavirus 16 and *Helicobacter pylori* infections might be causations for this type of carcinoma [14]–[16]. Although these two potential pathogens in the larynx have recently been reported, it is still unknown if single species or polymicrobial communities are involved in the pathogenesis.

The interest in the microbial characteristics in the throat has recently increased. However, progression towards detailed biodistribution of the laryngeal microbial community, both in healthy and sick patients has not been carefully explored. The current study was designed to investigate characteristics of microbial biology in laryngeal mucosa and analyze the abundant composition between laryngeal carcinoma patients and a control population. To our knowledge, this is the first study that has engaged barcoded pyrosequencing analysis of the 16S rRNA genes and a case-control design on laryngeal tissue samples to investigate throat microbiome.

## Materials and Methods

### Patients and Collection of Tissue Samples

Twenty-nine Laryngeal squamous cell carcinoma (LSCC) patients and 31 control patients with vocal cord polyps were enrolled in this study from January 2011 to March 2012. The laryngeal carcinoma patients had undergone total laryngeal resection and had a histopathologically confirmed diagnosis of LSCC. The control subjects were verified to be cancer-free, and also with no evidence of epithelial dysplasia. Patients with a history of antibiotic use in the previous 3 months or active bacterial or viral infections in other parts of the body were excluded from the study.

Tissue samples were collected in a laminar flow operation room to avoid contamination. Tumor tissues and normal mucosa tissues adjacent to tumor sites were taken from LSCC patients using separate surgical instruments to avoid cross contamination. The normal tissues located at least one centimeter from the tumor site. In the control group, tissue samples of vocal cord polyps were collected through a direct laryngoscope at the site of vocal cord of throat. All of the specimens were separately preserved in eppendorf tube without any medium at −80°C before further analysis.

Tumor stage was determined according to the International Union Against Cancer TNM classification system, 6th Edition [17]. Tumor volume measurement has been described previously [18]. The present study was approved by the Ethics Committee of the Eye, Ear, Nose, and Throat Hospital, Fudan University. All enrolled patients were informed and signed a written consent in accordance with the committee’s regulations.

### DNA Extraction and Polymerase Chain Reaction (PCR)

Genomic DNA was extracted from the samples using a QIAGEN DNeasy kit (QIAGEN, Hilden, Germany) according to the manufacturer’s instructions. A cocktail master mix containing lysozyme, mutanolysin, and lysostaphin (sigma – Aldrich, US) was added to each sample after proteinase K incubation. Stainless zirconia-silica-beads were also used to maximize DNA extraction from gram-positive bacteria. The 16S rRNA gene sequences were amplified V1 to V3 (Escherichia coli positions 27F ∼ 534R) by PCR. The primer sequences were as follows: 27F: 5′-*CCA TCT CAT CCC TGC GTG TCT CCG ACG ACT* - barcode- AGA GTT TGA TCC TGG CTC AG- 3′. The 534R: 5′-*CCT ATC CCC TGT GTG CCT TGG CAG TCT CAG* - ATT ACC GCG GCT GCT GG- 3′. The italic part was an adapter in primer respective, and a barcode fragment of 8 to 10 bases was used in the 27F primer (Sangon, Shanghai). PCR was performed under the following conditions: 95°C for 2 min, followed by 30 cycles of 95°C for 20 sec, 56°C for 30 sec, and 72°C for 5 min.

### Sequence Processing and Data Statistical Analyses

The products of amplicon of the 16S rRNA from different samples were mixed in equal ratios for pyrosequencing with the GS FLX platform. The prepared DNA samples were transformed into single stranded template DNA (sstDNA) libraries by using the GS DNA Library Preparation kit (Roche Applied Science). sstDNA libraries were clonally amplified in a bead immobilized form by using the GS emPCR kit (Roche Applied Science) and sequenced on the 454 Genome Sequencer FLX Titanium platform. Pyrosequencing was performed at the Chinese National Human Genome Sequencing Center (Shanghai, China).

The sequences containing the ambiguous base (N) were abandoned using customized perl scripts and only sequences with an average quality score ≥20 and min length ≥200 bp were included in the analysis. Denoising strategy was performed using Mothur based on Chris Quince’s PyroNoise algorithm [19].

UCLUST software was used to determine OTUs at a level of 97% similarity. Rarefaction curves, Shannon weaver, Simpson diversity indices, ACE, Chao1, and Good’s coverage were calculated by Mothur analysis [20] (v.1.27.0, http://www.mothur.org/wiki/Main_Page) at 3% distance level. The RDP classifier was used to assign sequences to phylogenetic taxonomy based on the Ribosomal Database Project [21], [22], and the sequences were assigned to the hierarchical taxa under the condition of bootstrap cut-off 80%. The statistical significance of abundance difference in microbial community composition between sample categories was determined by metastats (http://metastats.cbcb.umd.edu/). The hierarchical cluster of microbial communities and principal coordinates analysis (PCoA) were analyzed by UniFrac software and visualized by R software and package heatmap.2. Based on UniFrac sample distance, the statistical significance P value was computed by R package CrossMatch. OTU sample abundance matrix was scaled to mean equal zero and variance unit, sample distance was computed by R package vegan. Hierarchical cluster and principal component analysis (PCA) were implemented by R software and package heatmap.2. Partial least squares discrimination analysis (PLS-DA), leave one out cross validation (LOOCV), and multivariate analysis of variance (MANOVA) were applied to investigate the significant bacteria related to LSCC disease by R package mixOmics and R function manova.

## Results

### Clinical Characteristics of Subjects

Twenty-nine patients with laryngeal carcinoma (27 males and 2 females), and 31 control subjects (27 males and 4 females) were entered in this study. We collected 31 control mucosa samples from vocal cord polyps, 29 LSCC tissue samples and 27 corresponding normal tissues adjacent to tumors simultaneously (two LSCC specimens missed the matched normal tissues). The clinical parameters including age, sex, tumor location, tumor size, and T classification of subjects were shown in Table 1.

**Table 1:** Clinical characteristics of the subjects in current study.

	LSCC subjects	Control subjects
**Age**		
≤60	11	13
>60	18	18
**Gender**		
Male	27	27
Female	2	4
**Tumor location**		
Supraglottic	11	
Glottic	18	
**T classification**		
T1 and T2	12	
T3 and T4	17	
**Tumor sizes**		
≤2 cm^3^	13	
>2 cm^3^	16	

### Bacterial Community Profiles in Laryngeal Mucosa

The bacterial community in the laryngeal mucosa was investigated by barcoded pyrosequencing analysis of the 16S rRNA gene. After trimming primers and incomplete V3 fragments, 143,122 reads from the V1–V2 region were obtained. There were 63,688 reads from laryngeal tumor tissues, 35,654 reads from normal tissues adjacent to tumors, and 43,780 reads from normal mucosa of controls (Table 2). An average of 1645 reads for each sample with an average length of 320±28 bases was obtained. Determining operational taxonomic units (OTUs) at a level of 97% similarity, the sequencing reads were assigned to 3334 species level in tumor tissue, 2639 OTUs in normal tissues adjacent to tumors, and 2378 OTUs in controls. To evaluate the diversity and richness of these sequencing reads of species detected in samples, Rarefaction curves, Good’s coverage, ACE, Chao1, Shannon, and Simpson parameters were applied to estimate the quality (Table 2). The Rarefaction curves generated for unique, 1%, 3%, and 5% in the three groups, reached the saturation level at the 3% dissimilarity level (Figure S1). For the three groups of laryngeal communities investigated at the dissimilarity of 3%, the number of OTUs was close to the total number of OTUs estimated by Chao1 and ACE parameters. Good’s coverage was almost 98% for all sequences in these groups (Table 2).

**Table 2:** Sequencing data with richness and diversity estimation of bacterial taxa in three groups of laryngeal mucosa.

Group	Cut off	Reads	OTUs	ACE	95% CI		Chao	95% CI		Shannon	Simpson	Coverage
Tumor*	0.03	63688	3334	6942.95	6694.58	7209.67	5294.85	5032.67	5597.50	6.21	0.005	0.98
Adjacent§	0.03	35654	2639	5197.44	4992.29	5420.48	4205.07	3973.68	4476.57	6.05	0.007	0.97
Control†	0.03	43780	2378	4744.93	4548.94	4958.61	3720.68	3512.81	3966.64	5.42	0.015	0.98

* LSCC tumor group;

§ normal tissue adjacent to tumor group;

† control group. The operational taxonomic units (OTUs) were defined at 3% dissimilarity level. The coverage percentage (Good), richness estimators (ACE and Chao1) and diversity indices (Shannon and Simpson) were calculated by the Mothur analysis.

**Figure 1:**
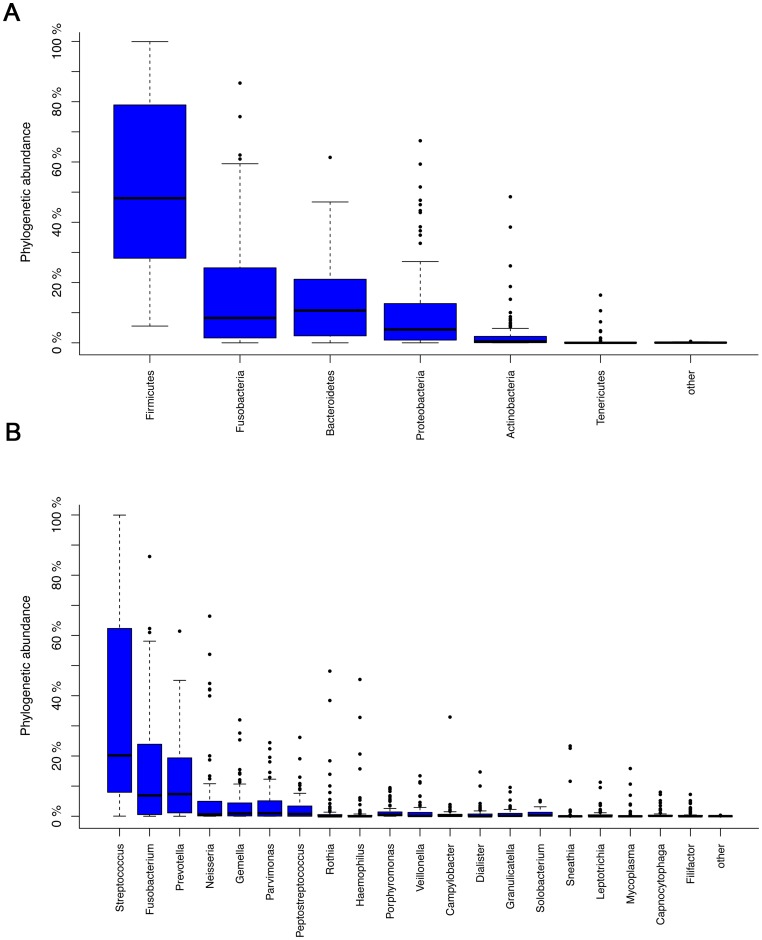
The major abundant communities of phyla (A) and genera (B) in laryngeal mucosa. Plotted values are mean sequence abundances in each phylum and genus.

**Figure 2:**
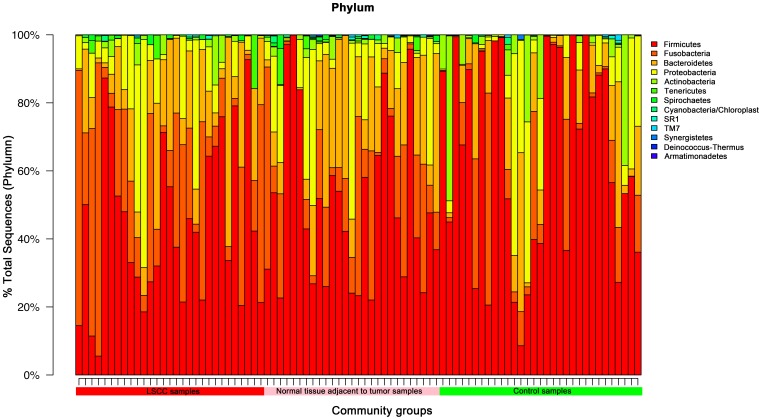
The detailing percentage of phyla with each laryngeal sample that involved the LSCC, normal tissue adjacent to tumor, and control groups. Color bars in the top-right corner indicate the phyla detected in the current study. Each color is an individual phylum, and each column is a laryngeal sample.

**Figure 3:**
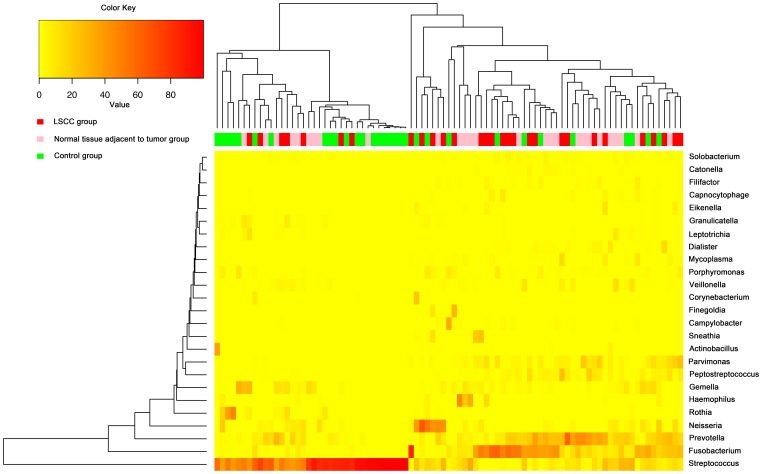
Heatmap of percentages of the main genera in the larynx of each individual based on 16S rRNA sequences. Complete linkage clustering of samples of the three groups (LSCC tumors, normal tissues adjacent to tumors, and controls) based on genera composition and abundance in communities. Each row is an individual genus, and each column is a laryngeal sample. Color key and color bars are indicated in the top-left corner.

For the overall bacterial community, 13 phyla were generated in laryngeal mucosa. The most prevalent communities of phyla with extreme ranges in diversity were the following: *Firmicutes* (54%, ranging span: 5.5–99.9%), *Fusobacteria* (17%, ranging span: 0–86.2%), *Bacteroidetes* (15%, ranging span: 0–61.5%), *Proteobacteria* (11%, ranging span: 0–67.1%), and *Actinobacteria* (3%, ranging span: 0–48.5%). Two-hundred-and-eighteen genera with a high ranging shift were detected, and the predominant genera presented were *Streptococcus* (36%, ranging span: 0.04–99.9%), *Fusobacterium* (15%, ranging span: 0–86.2%), *Prevotella* (12%, ranging span: 0–61.4%), *Neisseria* (6%, ranging span: 0–66.4%), and *Gemella* (4%, ranging span: 0–32.0%) (Figure 1). The main percentage of classes, orders and families of community in throat were shown in Figure S2. The detailing percentage of phyla and genera for each sample were detected (Figure 2 and Figure S3). A correlogram of laryngeal microbial community with three groups of LSCC tumors, normal tissue adjacent to tumor, and controls were analyzed and presented using heatmaps at the levels of phyla and genera (Figure 3 and Figure S4).

**Figure 4:**
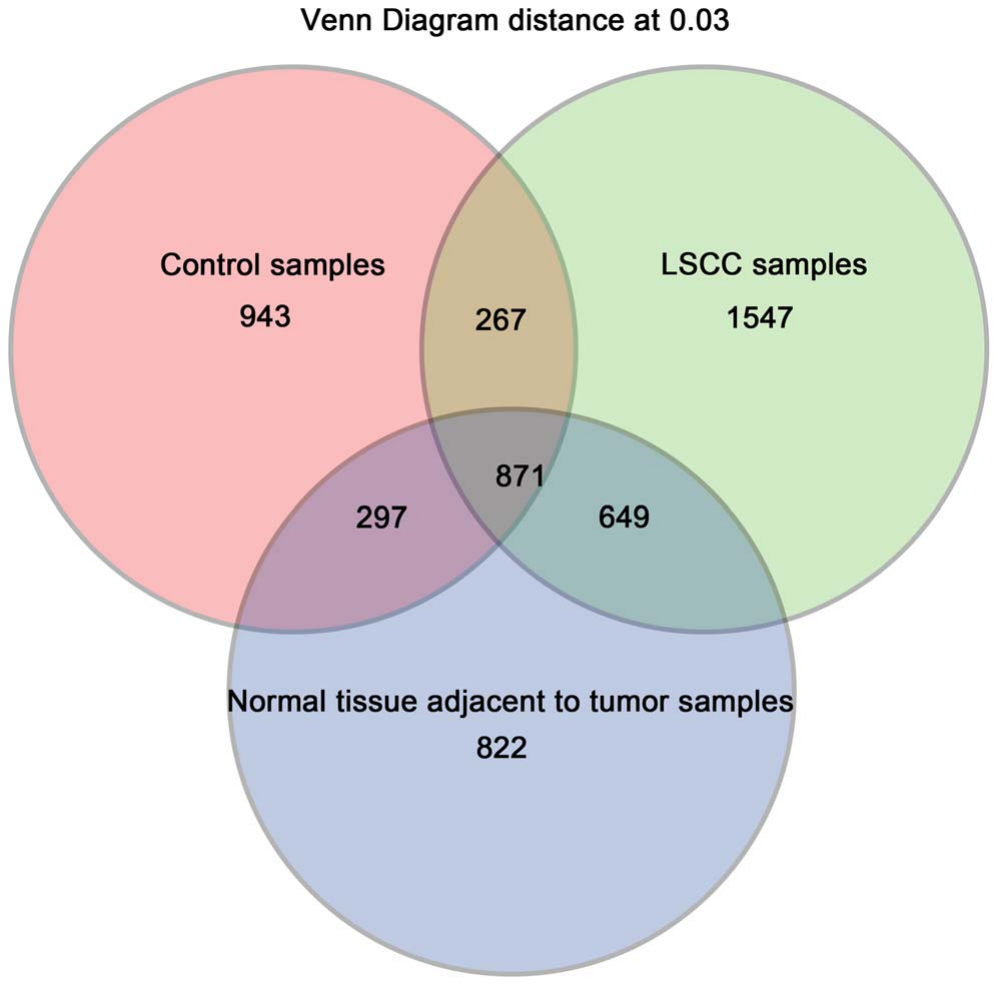
Venn diagrams for overlap of observed OTUs among the three groups. There were 871 species were shared in three groups, 1138 species were shared between the LSCC tumor and control groups, 1168 species were shared between the normal tissue adjacent to tumor and control groups, and 1520 species were shared between the LSCC tissue and normal tissue adjacent to tumor groups.

Defining OTUs at a level of 3% dissimilarity, the three groups shared a great degree of community similarity. A total of 871 OTUs were present and shared in these groups, 1138 OTUs species were shared between LSCC samples and controls, 1168 OTUs were shared between normal tissues adjacent to tumors and controls, and 1520 OTUs were shared between LSCC samples and normal tissues adjacent to tumors (Figure 4). The detailing taxon shared among groups of LSCC tumor samples, normal tissue adjacent to tumor samples, and control samples were also shown in Figure S5.

### The Microbial Biodiversity in Tumor and Normal Mucosa of Larynx

**Figure 5:**
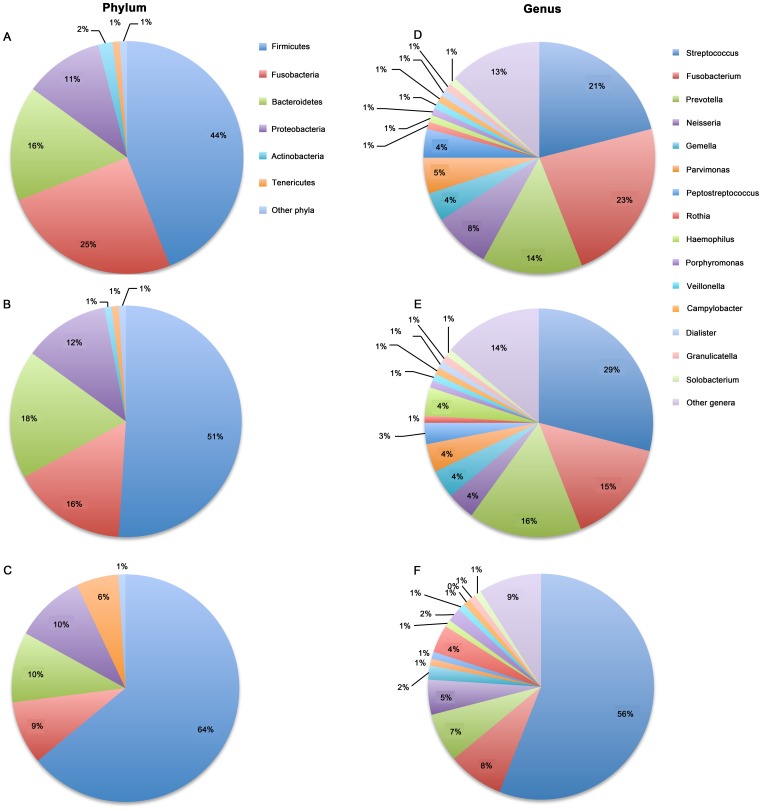
Relative abundance of the main bacterial phyla and genera in the larynx. Microbial profiles within the LSCC tumor (A), normal tissue adjacent to tumor (B), and control groups (C) at the level of phyla. Microbial profiles in the LSCC tumor (D), normal tissue adjacent to tumor (E), and control groups (F) at the level of genera. The values are mean sequence abundances in each group and each level. Color bars in the top-right of each column are indicated the main phyla and genera.

The microbial structures in the LSCC tumor, normal tissue adjacent to tumor, and control groups were significantly different (Table 3). The main phyla in the three groups mentioned above were *Firmicutes* (44%, 51%, and 64%, in the three groups, respectively), followed by *Fusobacteria* (25%, 16%, and 9%, in the three groups, respectively), *Bacteroidetes* (16%, 18%, and 10%, in the three groups, respectively), *Proteobacteria* (11%, 12%, and 10%, in the three groups, respectively), and *Actinobacteria* (2%, 1%, and 6%, in the three groups, respectively). The dominant communities of genera in laryngeal mucosa were *Streptococcus* (21%, 29%, and 56%, in the three groups, respectively), *Fusobacterium* (23%, 15%, and 8%, in the three groups, respectively), *Prevotella* (14%, 16%, and 7%, in the three groups, respectively), *Neisseria* (8%, 4%, and 5%, in the three groups, respectively), and *Gemella* (4%, 4%, and 2%, in the three groups, respectively) (Figure 5 and Table 3). When the bacterial communities of the LSCC tumor and normal tissue adjacent to tumor groups were combined as the laryngeal cancer group and compared to the control group, there were 6 phyla and 26 genera that were significantly different between the two groups (*p*<0.05) (Table S1).

**Table 3:** The comparison of bacterial community in LSCC group, normal tissue adjacent to tumor group, and control group at the levels of phyla and genera.

	Cancer – Adjacent	Cancer – Control	Adjacent – Control
	*p* value*	*p* value^§^	*p* value^†^
**Phyla**			
*Firmicutes*	0.37	0.004	0.051
*Fusobacteria*	0.09	0.001	0.14
*Bacteroidetes*	0.59	0.11	0.04
*Proteobacteria*	0.81	0.73	0.56
*Actinobacteria*	0.78	0.03	0.02
**Genera**			
*Streptococcus*	0.33	<0.001	0.002
*Fusobacterium*	0.09	0.002	0.17
*Prevotella*	0.41	0.04	0.005
*Neisseria*	0.33	0.46	0.78
*Gemella*	0.98	0.17	0.15

* The comparison of bacterial composition between LSCC tumor group and normal tissue adjacent to tumor group; ^§^ the comparison of bacterial composition between LSCC tumor group and control group; ^†^ the comparison of bacterial composition of normal tissue adjacent to tumor group and control group. *p* value were analyzed from Student t-test and matched to Figure 5.

There were no significantly different bacterial communities between the supraglottic and glottic tumor groups. Comparing the groups with tumor size of less than or equal to 2 cm (≤2 cm) and greater than 2 cm (>2 cm), we observed that the genera of *Prevotella* (7.2% in ≤2 cm group and 18.8% in >2 cm, respectively, *p* = 0.026) and *Peptostreptococcus* (1.3% in ≤2 cm group and 5.8% in >2 cm group, respectively, *p* = 0.019) were significantly different between the two groups. Analyzing the bacterial distribution in different age groups, we found the *Oribacterium* genus was more abundant in subjects 60 years of age or younger (1.2%) compared to subjects older than 60 years in age (0.02%, *p* = 0.047). Investigating the community structure in T classification, we detected that the genera of *Prevotella* (4.9% in T1 and T2 group and 19.8% in T3 and T4 group, respectively, *p* = 0.004) and *Solobacterium* (0.1% in T1 and T2 group and 1% in T3 and T4 group, respectively, *p* = 0.009) were significantly different.

**Figure 6:**
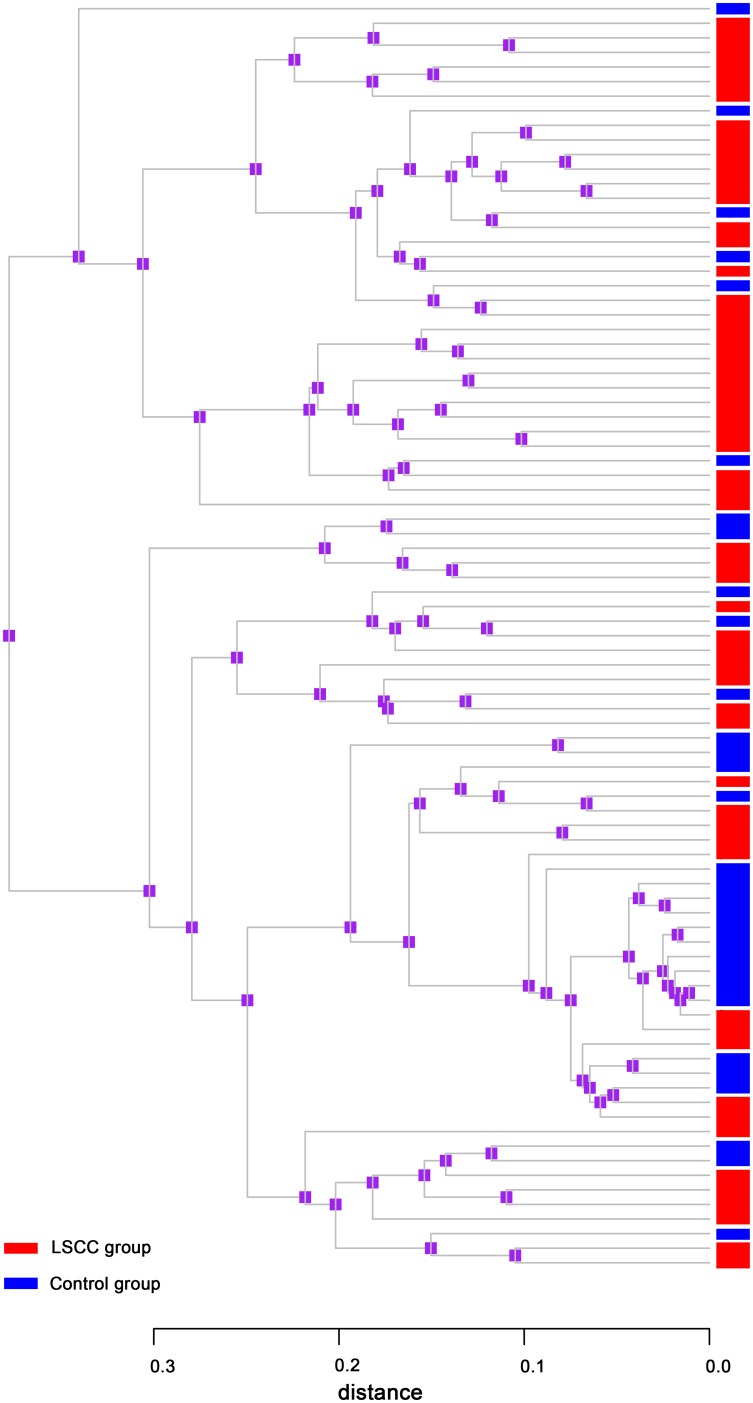
Weighted UniFrac tree from cluster analyses with laryngeal cancer patients and controls. The scale bar indicates a weighted UniFrac distance from 0 to 0.3. Samples from the control and cancer groups tended to cluster together referring to the different healthy conditions. Color bars of the lower -left indicate the LSCC and control groups.

**Figure 7:**
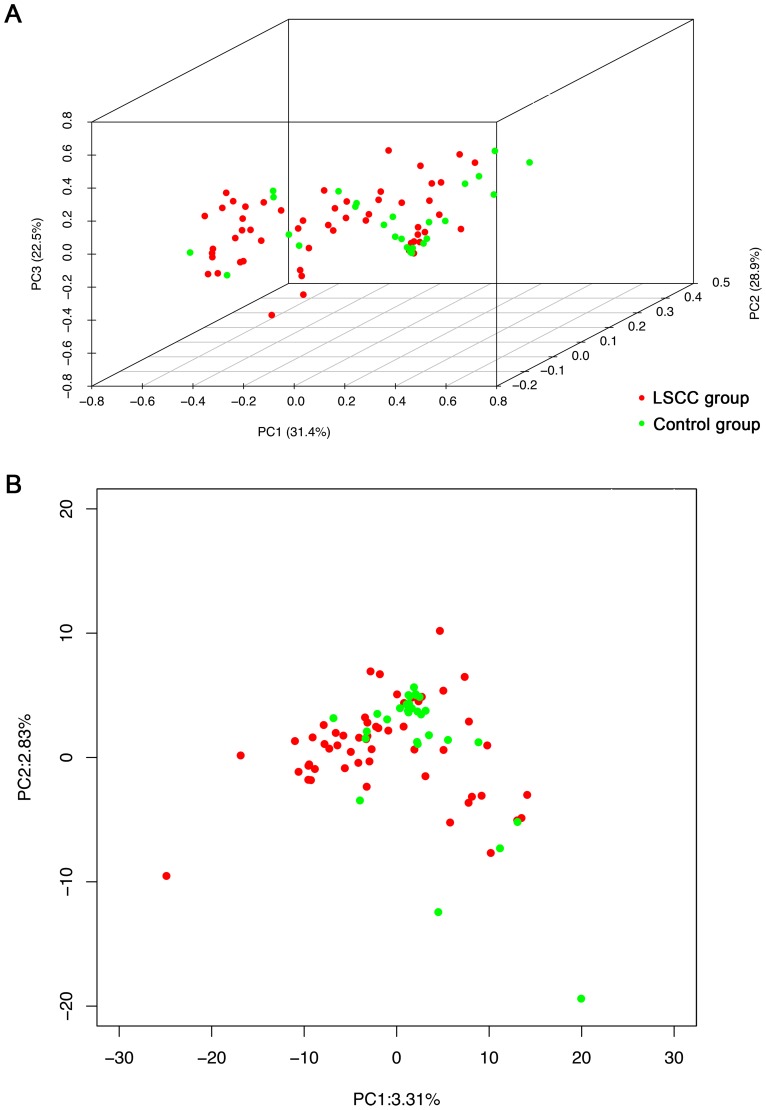
Bacterial diversity clustering by laryngeal cancer patients and controls. Principal coordinates analysis (*p* = 0.006) and principal component analysis (*p = *0.01) of the LSCC (red) population and control subjects (green) based on community composition and abundance. Each symbol represents a sample. The variances explained by the analyses were indicated in parentheses on the axes. Laryngeal cancer and control samples almost group together according to the different healthy conditions.

Comparing the overall bacterial community by the UniFrac, a phylogenetic tree based on metrics ranging from zero (distance between same communities) to one (distance between totally distinct communities with no shared ancestry) was generated [23]. The connection clustering of the samples were based on the species’ structures and abundance of laryngeal bacterial communities that defined the community of the two groups. We observed that samples in the LSCC and control groups formed large sections that were obviously separated (Figure 6). The bacterial communities from laryngeal cancer and control groups were determined and graphically presented with the abundance of each phylotype represented by a block, and the hierarchical cluster of microbial communities of two groups were also involved in this analyses (Figure S6). We then applied PCoA and PCA analyses to confirm the findings that the microbiote structure of LSCC population and controls were significantly different (*p* = 0.006 from PCoA and *p = *0.01 from PCA, respectively) (Figure 7). After PLS-DA, LOOCV and MANOVA analyses, we found that the composition of 15 genera were significantly different in the two groups, and may be associated with laryngeal carcinoma (*p*<0.001). For example, the abundance of *Fusobacterium*, *Prevotella*, and *Gemella* were higher in the LSCC group (19.1%, 15.0%, and 4.4%, respectively) than in controls (7.9%, 6.9%, and 2.2%, respectively). The prevalence of *Streptococcus* and *Rothia* were higher in the control population (56.1%and 4.2%, respectively) compared with the LSCC population (25.1% and 0.8%, respectively) (Table 4) (Figure S7).

**Table 4:** The bacterial composition of 15 genera were significantly different between the laryngeal cancer and control groups.

	Cancer group	Control group	*p* value*
Genera	%	%	
*Rothia*	0.8	4.2	<0.001
*Streptococcus*	25.1	56.1	
*Fusobacterium*	19.1	7.9	
*Prevotella*	15.0	6.9	
*Gemella*	4.4	2.2	
*Granulicatella*	0.9	0.5	
*Peptostreptococcus*	3.6	0.6	
*Parvimonas*	4.6	1.3	
*Peptostreptococcaceae incertae sedis*	0.5	0.04	
*Porphyromonas*	1.0	2.0	
*Catonella*	0.7	0.2	
*Treponema*	0.5	0.1	
*Bulleidia*	0.02	0.1	
*Selenomonas*	0.5	0.04	
*Burkholderia*	0.03	0.003	

* PLS-DA, LOOCV and MANOVA analyses were applied to investigate the bacterial composition between the laryngeal cancer and control groups. Fifteen genera probably were related to LSCC disease.

## Discussion

The present findings revealed bacterial composition in laryngeal mucosa. The estimation parameters corroborated indicated that the 16S rRNA sequences investigated in this study could represent the natural laryngeal bacterial profiles. Two-hundred-and-eighteen genera from 13 phyla were detected in the larynx. We observed that the predominant communities of phyla in the larynx were *Firmicutes*, *Fusobacteria*, *Bacteroidetes*, *Proteobacteria*, and *Actinobacteria*. The leading genera presented in laryngeal mucosa were *Streptococcus, Fusobacterium, Prevotella, Neisseria,* and *Gemella*. The microbial structure in groups of LSCC tumor, normal tissue adjacent to tumor, and normal laryngeal mucosa were highly diverse. We found that numerous bacterial composition were significantly different between laryngeal cancer patients and control subjects in throat. Furthermore, fifteen genera may be correlated to laryngeal carcinoma such as *Fusobacterium*, *Prevotella,* and *Streptococcus*.

The larynx is part of the respiratory system and is also adjacent to the oral cavity, nose, nasopharynx, oropharynx, trachea and lung; however, the ecological profiles of laryngeal mucosa are different from these adjacent sites [5], [8], [10], [24]–[27]. It is reported that the microbial composition is different in specific anatomical niches, and there are likely antagonistic effects between these microorganisms [26]. Our current findings about the laryngeal microbiome have confirmed the previous suggestions further. These data reveal the different biological dynamics among these anatomic sites despite the fact that they are connected.

Pyrosequencing has been suggested to overestimate diversity due to sequencing errors and short sequence lengths. It is reported that high stringent quality-based trimming and clustering thresholds ≤97% is the simplest and least computationally intensive measures to confirm that 16S pyrotag analyses provide accurate, high sensitivity phylogenetic profiling of microbiota currently [28]. V1– V2 are the hypervariable regions, and there is massively taxonomic classification information in these parts [29]. In recent, we can only obtain part of 16S rRNA gene information because of the technical limitation of sequencing platform. The V1– V2 region is also widely used to analyze in taxology for microbiota in different body sites [29]–[34]. Therefore, investigating the pyrosequencing data in appropriately method probably can generate the objective results.

There is an emerging concept that the microbial community as a unit of pathogenicity may cause many endogenous diseases [35]–[37]. Multiple communities joining together may give rise to the same disease outcomes [11]. In our study, the assembly of communities of several genera was significantly different between laryngeal cancer patients and the control population. This may explain why these species, found in various prevalence in people, may have an important ecological role in determining the pathology or protecting from disease. For these different communities between the two groups, several genera were not the predominant group in the larynx. It is likely that these genera of bacteria do not have crucial roles in this environment. But it has been widely suggested that low level microorganisms may serve critical roles in human ecological niches and may have had a profound impact on shaping the microenvironment over time by providing a nearly inexhaustible source of genomic innovation and may hold the potential to become a major portion in response to changes in environmental conditions [7], [35], [38]–[40].

In current study, we found the assembly of the 15 genera was highly different between the laryngeal cancer and control groups, and may be related to laryngeal carcinoma. For instance, *Fusobacterium* and *Prevotella* were more abundant in the laryngeal cancer population. Conversely, the *Streptococcus* was more prevalent in control laryngeal mucosa.

The microbiota could profoundly affect many aspects of host physiology, such as activating immune system [41], [42], regulating metabolism [31], [41], and promoting cancer [43]. It is reported that the altered microbial composition and induced the expansion of microorganisms with genotoxic capabilities can promote tumorigenesis in mice intestine [43], [44]. It has been widely accepted that transition from a normal epithelium to laryngeal carcinoma is a lengthy, comprehensive and multistage process [45]. There are numerous complex interplays between microbiota and the initiation and progression of carcinoma, such as through Toll-like receptor signaling probably [46].

We hypothesized that the microbiota and disease might interact as both cause and effect in different stages of disease. Some bacteria might as a pathogenesis to induce the throat microenviroment to dysbiosis situation primarily. It is suggested that *Fusobacterium* and *Prevotella* initiate the formations of biofilms and stimulate cytokines/chemokines that contribute to disease development [47], [48]. However, *Streptococcus* may have an antagonistic role to affect *Fusobacterium* and *Prevotella* through energy metabolism and nutrient transfer, such as the phosphoenolpyruvate-dependent phosphotransferase system [49]. In the biofilm niche, these microorganisms co-exist to act both synergistically and competitively [7], [11]. These complex interactions involving attachment, recruitment, maturation and detachment may occur in these communities [49]. It has been reported that a number of the biofilms appeared to inhibit either the normal basal level of message, basal level of translation and secretion, and/or degradation of various mediators in this biofilm model system [47], [48]. Hence, disturbed microbial ecology by polymicrobial effects might be required for the initiation of disease.

Furthermore, the shift of the bacteria communities probably also is a result of the disease in advanced tumor stages. The *Prevotella* was more prevalent in T3 and T4 stages and tumors greater than 2 cm in LSCC. *Prevotella* and *Fusobacterium* are anaerobe and highly infected in solid cancer patients [50]. The solid tumors grow rapidly and stimulate angiogenesis to promote the formation of new blood vessels, but the newly formed vessels fail to provide adequate oxygen to the all tumor cells [51]. Finally, the multiple regions of hypoxia and anoxia form within the tumors. Hypoxic and anoxic regions are often near to regions of necrosis and provide a selective and suitable microenvironment for the growth of anaerobic bacteria [50], [51]. *Prevotella* and *Fusobacterium* are becoming more abundant. Finally, the more prevalent percentage of *Fusobacterium* and *Prevotella* probably lead to the worse microecology and then contribute the development and progression of disease in throat.

This hypothesis need further research to confirm.

### Conclusions

We detected 218 genera from 13 phyla in laryngeal mucosa, and these microorganisms may interact in the development and maintenance of healthy homeostasis in the laryngeal ecological niche. The microbial structures in laryngeal cancer and control subjects were significantly different. Fifteen genera may be associated to laryngeal carcinoma. The bacteria communities and laryngeal cancer probably interact as both cause and effect in different stages. The disruption of this bio-ecological niche might be a risk factor for laryngeal carcinoma.

## Supporting Information

Figure S1 **Evaluation of the bacterial community diversity and richness in laryngeal mucosa.** Rarefaction curves of the LSCC tumor (A), normal tissue adjacent to tumor (B), and control groups (C) generated with unique, 1%, 3%, and 5%, reaching the saturation level at the 3% dissimilarity level. Shannon curves of the LSCC tumor (D), normal tissue adjacent to tumor (E), and control groups (F) were obtained. The vertical axis indicates the Shannon diversity, and the horizontal axis shows the sequence number.
(TIF)

Figure S2 **The major abundant communities in the larynx at the level of classes (A), orders (B), families (C).** Plotted values are mean sequence abundances in each class, order, and family.
(TIF)

Figure S3 **The detailing percentage of the main genera with each laryngeal sample that included LSCC tumor group, normal tissue adjacent to tumor group, and control group.** Each color is an individual genus, and each column is a laryngeal tissue sample. Color bars in the right indicate the genera detected in the current study.
(TIF)

Figure S4 **Heatmap of the percentage of the phyla in the larynx of each individual.** Complete linkage clustering of samples of the three groups (LSCC tumor, normal tissue adjacent to tumor, and control groups) based on phyla composition and abundance in communities. Each row is an individual phylum, and each column is a laryngeal sample. Color key and color bars are presented in the top-left corner.
(TIF)

Figure S5 **Venn diagrams for overlap among the three groups (LSCC tumor, normal tissue adjacent to tumor, and control groups) at the levels of phyla (A), classes (B), orders (C), families (D), and genera (E).**
(TIF)

Figure S6 **Correlogram of each laryngeal sample.** The correlogram was build by community composition and abundance in laryngeal cancer patients and controls. Some samples tended to group together according to cancer group or control status. Color key and color bars are indicated in the top-left corner.
(TIF)

Figure S7 **Heatmap of the abundance of the divergent genera in the laryngeal cancer patients and controls.** The composition and prevalence of 15 genera were significantly different between laryngeal cancer group and controls investigated by PLS-DA, LOOCV and MANOVA analyses. Color key and color bars are indicated in the top-left corner.
(TIF)

Table S1 **The community composition difference between laryngeal cancer and control groups.**
(DOCX)

## References

[1] Weinstock GM (2012) Genomic approaches to studying the human microbiota. Nature 489: 250–256. 22972298 10.1038/nature11553 PMC3665339

[2] Lozupone CA, Stombaugh JI, Gordon JI, Jansson JK, Knight R (2012) Diversity, stability and resilience of the human gut microbiota. Nature 489: 220–230. 22972295 10.1038/nature11550 PMC3577372

[3] Ursell LK, Metcalf JL, Parfrey LW, Knight R (2012) Defining the human microbiome. Nutr Rev 70 Suppl 1S38–44. 22861806 10.1111/j.1753-4887.2012.00493.x PMC3426293

[4] Dethlefsen L, McFall-Ngai M, Relman DA (2007) An ecological and evolutionary perspective on human-microbe mutualism and disease. Nature 449: 811–818. 17943117 10.1038/nature06245 PMC9464033

[5] Peterson J, Garges S, Giovanni M, McInnes P, Wang L, et al (2009) The NIH Human Microbiome Project. Genome Res 19: 2317–2323. 19819907 10.1101/gr.096651.109 PMC2792171

[6] James WP, Garza C (2012) Summary of the 24(th) Marabou Symposium: Nutrition and the human microbiome. Nutr Rev 70 Suppl 1S87–94. 22861809 10.1111/j.1753-4887.2012.00497.x

[7] Walker A (2007) Say hello to our little friends. Nat Rev Microbiol 5: 572–573. 17633442 10.1038/nrmicro1720

[8] Huse SM, Ye Y, Zhou Y, Fodor AA (2012) A Core Human Microbiome as Viewed through 16S rRNA Sequence Clusters. PLoS One 7: e34242 doi: 34210.31371/journal.pone.0034242 22719824 10.1371/journal.pone.0034242 PMC3374614

[9] Widdicombe J, Kamath S (2004) Acute cough in the elderly: aetiology, diagnosis and therapy. Drugs Aging 21: 243–258. 15012170 10.2165/00002512-200421040-00003 PMC7100077

[10] Goddard AF, Staudinger BJ, Dowd SE, Joshi-Datar A, Wolcott RD, et al (2012) Direct sampling of cystic fibrosis lungs indicates that DNA-based analyses of upper-airway specimens can misrepresent lung microbiota. Proc Natl Acad Sci U S A 109: 13769–13774. 22872870 10.1073/pnas.1107435109 PMC3427132

[11] Costello EK, Stagaman K, Dethlefsen L, Bohannan BJ, Relman DA (2012) The application of ecological theory toward an understanding of the human microbiome. Science 336: 1255–1262. 22674335 10.1126/science.1224203 PMC4208626

[12] Parkin DM, Bray F, Ferlay J, Pisani P (2005) Global Cancer Statistics, 2002. CA Cancer J Clin 55: 74–108. 15761078 10.3322/canjclin.55.2.74

[13] Rudolph E, Dyckhoff G, Becher H, Dietz A, Ramroth H (2011) Effects of tumour stage, comorbidity and therapy on survival of laryngeal cancer patients: a systematic review and a meta-analysis. Eur Arch Otorhinolaryngol 268: 165–179. 20957488 10.1007/s00405-010-1395-8

[14] Gong H, Shi Y, Zhou L, Tao L, Shi Y, et al (2012) Helicobacter pylori infection of the larynx may be an emerging risk factor for laryngeal squamous cell carcinoma. Clin Transl Oncol 14: 905–910. 22855167 10.1007/s12094-012-0879-y

[15] Marur S, D’Souza G, Westra WH, Forastiere AA (2010) HPV-associated head and neck cancer: a virus-related cancer epidemic. Lancet Oncol 11: doi: 10.1016/S1470-2045(1010)70017-70016. Epub 72010 May 70015 10.1016/S1470-2045(10)70017-6 PMC5242182 20451455

[16] Zhuo XL, Wang Y, Zhuo WL, Zhang XY (2008) Possible association of Helicobacter pylori infection with laryngeal cancer risk: an evidence-based meta-analysis. Arch Med Res 39: 625–628. 18662596 10.1016/j.arcmed.2008.04.008

[17] Sobin LH, Wittekind CH (2002) International Union Against Cancer (UICC): TNM classification of malignant tumors: Wiley.

[18] Tomayko MM, Reynolds CP (1989) Determination of subcutaneous tumor size in athymic (nude) mice. Cancer Chemother Pharmacol 24: 148–154. 2544306 10.1007/BF00300234

[19] Quince C, Lanzen A, Curtis TP, Davenport RJ, Hall N, et al (2009) Accurate determination of microbial diversity from 454 pyrosequencing data. Nat Methods 6: 639–641. 19668203 10.1038/nmeth.1361

[20] Schloss PD, Westcott SL, Ryabin T, Hall JR, Hartmann M, et al (2009) Introducing mothur: open-source, platform-independent, community-supported software for describing and comparing microbial communities. Appl Environ Microbiol 75: 7537–4751. 19801464 10.1128/AEM.01541-09 PMC2786419

[21] Cole JR, Wang Q, Cardenas E, Fish J, Chai B, et al (2009) The Ribosomal Database Project: improved alignments and new tools for rRNA analysis. Nucleic Acids Res 37: D141–145. 19004872 10.1093/nar/gkn879 PMC2686447

[22] Wang Q, Garrity GM, Tiedje JM, Cole JR (2007) Naive Bayesian classifier for rapid assignment of rRNA sequences into the new bacterial taxonomy. Appl Environ Microbiol 73: 5261–5267. 17586664 10.1128/AEM.00062-07 PMC1950982

[23] Koren O, Spor A, Felin J, Fåk F, Stombaugh J, et al (2011) Human oral, gut, and plaque microbiota in patients with atherosclerosis. Proc Natl Acad Sci U S A 108: 4592–4598. 20937873 10.1073/pnas.1011383107 PMC3063583

[24] Bogaert D, Keijser B, Huse S, Rossen J, Veenhoven R, et al (2011) Variability and diversity of nasopharyngeal microbiota in children: a metagenomic analysis. Plos one 6: e17035. 21386965 10.1371/journal.pone.0017035 PMC3046172

[25] Yamanaka W, Takeshita T, Shibata Y, Matsuo K, Eshima N, et al. (2012) Compositional Stability of a Salivary Bacterial Population against Supragingival Microbiota Shift following Periodontal Therapy. PLoS One 7: doi: 10.1371/journal.pone.0042806. Epub 0042012 Aug 0042816 10.1371/journal.pone.0042806 PMC3420916 22916162

[26] Lemon KP, Klepac-Ceraj V, Schiffer HK, Brodie EL, Lynch SV, et al.. (2010) Comparative analyses of the bacterial microbiota of the human nostril and oropharynx. MBio 1. 10.1128/mBio.00129-10 PMC2925076 20802827

[27] Pragman AA, Kim HB, Reilly CS, Wendt C, Isaacson RE (2012) The lung microbiome in moderate and severe chronic obstructive pulmonary disease. Plos one 7: e47305. 23071781 10.1371/journal.pone.0047305 PMC3469539

[28] Kunin V, Engelbrektson A, Ochman H, Hugenholtz P (2010) Wrinkles in the rare biosphere: pyrosequencing errors can lead to artificial inflation of diversity estimates. Environ Microbiol 12: 118–123. 19725865 10.1111/j.1462-2920.2009.02051.x

[29] Upadhyay V, Poroyko V, Kim TJ, Devkota S, Fu S, et al (2012) Lymphotoxin regulates commensal responses to enable diet-induced obesity. Nat Immunol 13: 947–953. 22922363 10.1038/ni.2403 PMC3718316

[30] Hand D, Wallis C, Colyer A, Penn CW (2013) Pyrosequencing the canine faecal microbiota: breadth and depth of biodiversity. Plos one 8: e53115. 23382835 10.1371/journal.pone.0053115 PMC3561364

[31] Hashimoto T, Perlot T, Rehman A, Trichereau J, Ishiguro H, et al (2012) ACE2 links amino acid malnutrition to microbial ecology and intestinal inflammation. Nature 487: 477–481. 22837003 10.1038/nature11228 PMC7095315

[32] Yamanaka W, Takeshita T, Shibata Y, Matsuo K, Eshima N, et al (2012) Compositional stability of a salivary bacterial population against supragingival microbiota shift following periodontal therapy. Plos one 7: e42806. 22916162 10.1371/journal.pone.0042806 PMC3420916

[33] Koren O, Spor A, Felin J, Fak F, Stombaugh J, et al (2011) Human oral, gut, and plaque microbiota in patients with atherosclerosis. Proc Natl Acad Sci U S A 108 Suppl 14592–4598. 20937873 10.1073/pnas.1011383107 PMC3063583

[34] Nasidze I, Li J, Schroeder R, Creasey JL, Li M, et al (2011) High diversity of the saliva microbiome in Batwa Pygmies. Plos one 6: e23352. 21858083 10.1371/journal.pone.0023352 PMC3156759

[35] Li L, Hsiao WW, Nandakumar R, Barbuto SM, Mongodin EF, et al (2010) Analyzing endodontic infections by deep coverage pyrosequencing. J Dent Res 89: 980–984. 20519493 10.1177/0022034510370026 PMC3318071

[36] Santos AL, Siqueira JF Jr, Rocas IN, Jesus EC, Rosado AS, et al (2011) Comparing the bacterial diversity of acute and chronic dental root canal infections. Plos one 6: e28088. 22132218 10.1371/journal.pone.0028088 PMC3221700

[37] Siqueira JF Jr, Rocas IN, Debelian GJ, Carmo FL, Paiva SS, et al (2008) Profiling of root canal bacterial communities associated with chronic apical periodontitis from Brazilian and Norwegian subjects. J Endod 34: 1457–1461. 19026873 10.1016/j.joen.2008.08.037

[38] Huse SM, Dethlefsen L, Huber JA, Mark Welch D, Relman DA, et al (2008) Exploring microbial diversity and taxonomy using SSU rRNA hypervariable tag sequencing. PLoS Genet 4: e1000255 doi: 1000210.1001371/journal.pgen.1000255 19023400 10.1371/journal.pgen.1000255 PMC2577301

[39] Sogin ML, Morrison HG, Huber JA, Mark Welch D, Huse SM, et al (2006) Microbial diversity in the deep sea and the underexplored “rare biosphere”. Proc Natl Acad Sci U S A 103: 12115–12120. 16880384 10.1073/pnas.0605127103 PMC1524930

[40] Zaura E, Keijser BJ, Huse SM, Crielaard W (2009) Defining the healthy “core microbiome” of oral microbial communities. BMC Microbiol 9: 259. 20003481 10.1186/1471-2180-9-259 PMC2805672

[41] Kau AL, Ahern PP, Griffin NW, Goodman AL, Gordon JI (2011) Human nutrition, the gut microbiome and the immune system. Nature 474: 327–336. 21677749 10.1038/nature10213 PMC3298082

[42] Kelly D, Mulder IE (2012) Microbiome and immunological interactions. Nutr Rev 70 Suppl 1S18–30. 22861803 10.1111/j.1753-4887.2012.00498.x

[43] Arthur JC, Perez-Chanona E, Muhlbauer M, Tomkovich S, Uronis JM, et al (2012) Intestinal inflammation targets cancer-inducing activity of the microbiota. Science 338: 120–123. 22903521 10.1126/science.1224820 PMC3645302

[44] Kahrstrom CT (2012) Bacterial pathogenesis: E. coli claims the driving seat for cancer. Nat Rev Microbiol 10: 670. 10.1038/nrmicro2878 22926206

[45] Gale N, Michaels L, Luzar B, Poljak M, Zidar N, et al (2009) Current review on squamous intraepithelial lesions of the larynx. Histopathology 54: 639–656. 18752537 10.1111/j.1365-2559.2008.03111.x

[46] Round JL, Lee SM, Li J, Tran G, Jabri B, et al (2011) The Toll-like receptor 2 pathway establishes colonization by a commensal of the human microbiota. Science 332: 974–977. 21512004 10.1126/science.1206095 PMC3164325

[47] Peyyala R, Kirakodu SS, Novak KF, Ebersole JL (2012) Oral microbial biofilm stimulation of epithelial cell responses. Cytokine 58: 65–72. 22266273 10.1016/j.cyto.2011.12.016 PMC4091036

[48] Peyyala R, Kirakodu SS, Novak KF, Ebersole JL (2013) Oral Epithelial Cell Responses to Multispecies Microbial Biofilms. J Dent Res. 10.1177/0022034512472508 PMC3576995 23300185

[49] Hendrickson EL, Wang T, Dickinson BC, Whitmore SE, Wright CJ, et al (2012) Proteomics of Streptococcus gordonii within a model developing oral microbial community. BMC Microbiol 12: 211. 22989070 10.1186/1471-2180-12-211 PMC3534352

[50] El-Sharif A, Elkhatib WF, Ashour HM (2012) Nosocomial infections in leukemic and solid-tumor cancer patients: distribution, outcome and microbial spectrum of anaerobes. Future Microbiol Dec: 1423–1429. 10.2217/fmb.12.125 23231490

[51] Tirandaz H, Mohammadi E (2013) Efficient tumor targeting by anaerobic butyrate-producing bacteria. Med Hypotheses 80: 675–678. 23410499 10.1016/j.mehy.2013.01.024

