# Supplementary figures and images for: The Composition of Microbiome in Larynx and the Throat Biodiversity between Laryngeal Squamous Cell Carcinoma Patients and Control Population
Source: PLoS One. 2013 Jun 18;8(6):e66476. doi: 10.1371/journal.pone.0066476 (PMC3688906; doi:10.1371/journal.pone.0066476)

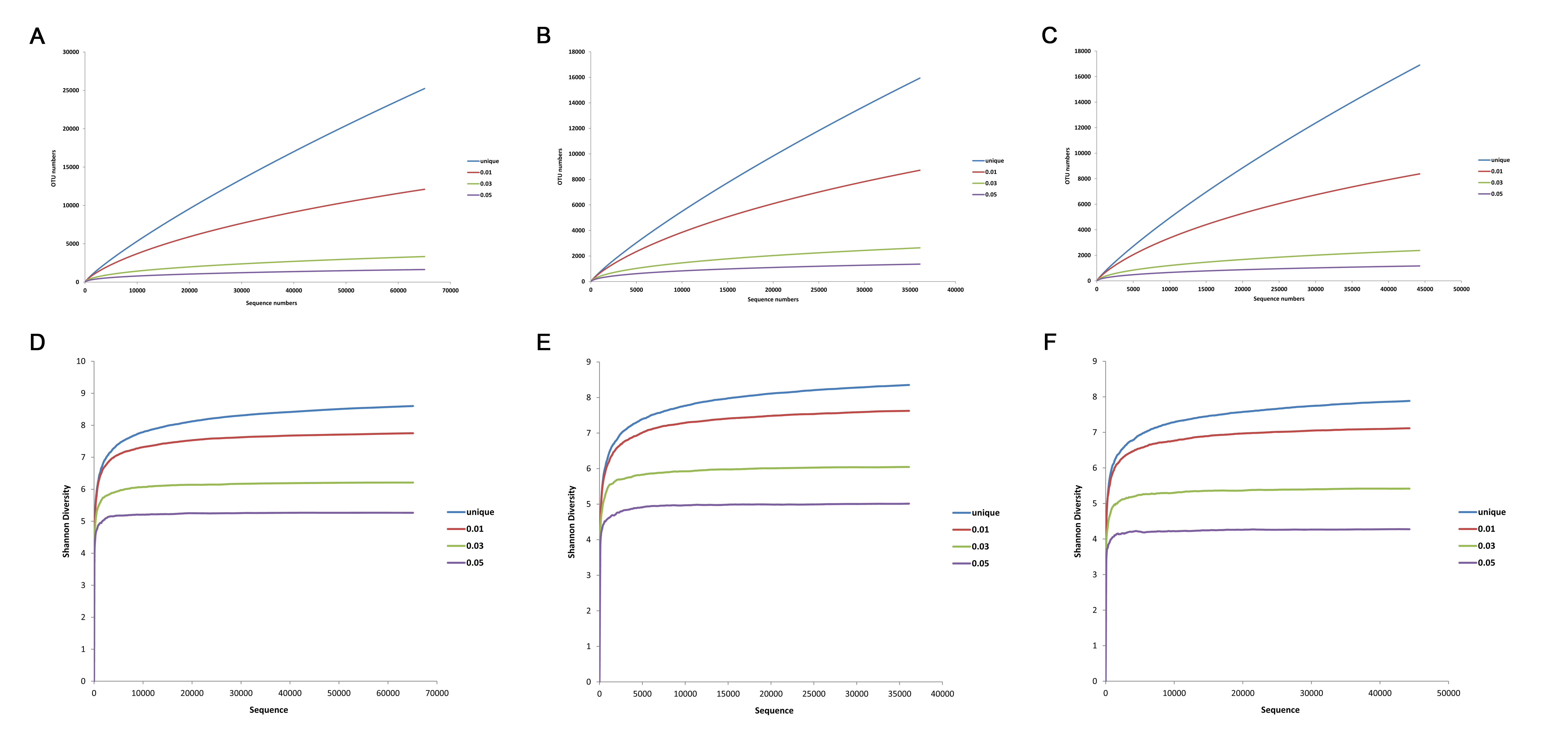

Supplement: Figure S1 — Evaluation of the bacterial community diversity and richness in laryngeal mucosa. Rarefaction curves of the LSCC tumor (A), normal tissue adjacent to tumor (B), and control groups (C) generated with unique, 1%, 3%, and 5%, reaching the saturation level at the 3% dissimilarity level. Shannon curves of the LSCC tumor (D), normal tissue adjacent to tumor (E), and control groups (F) were obtained. The vertical axis indicates the Shannon diversity, and the horizontal axis shows the sequence number. (TIF) [file pone.0066476.s001.tif]

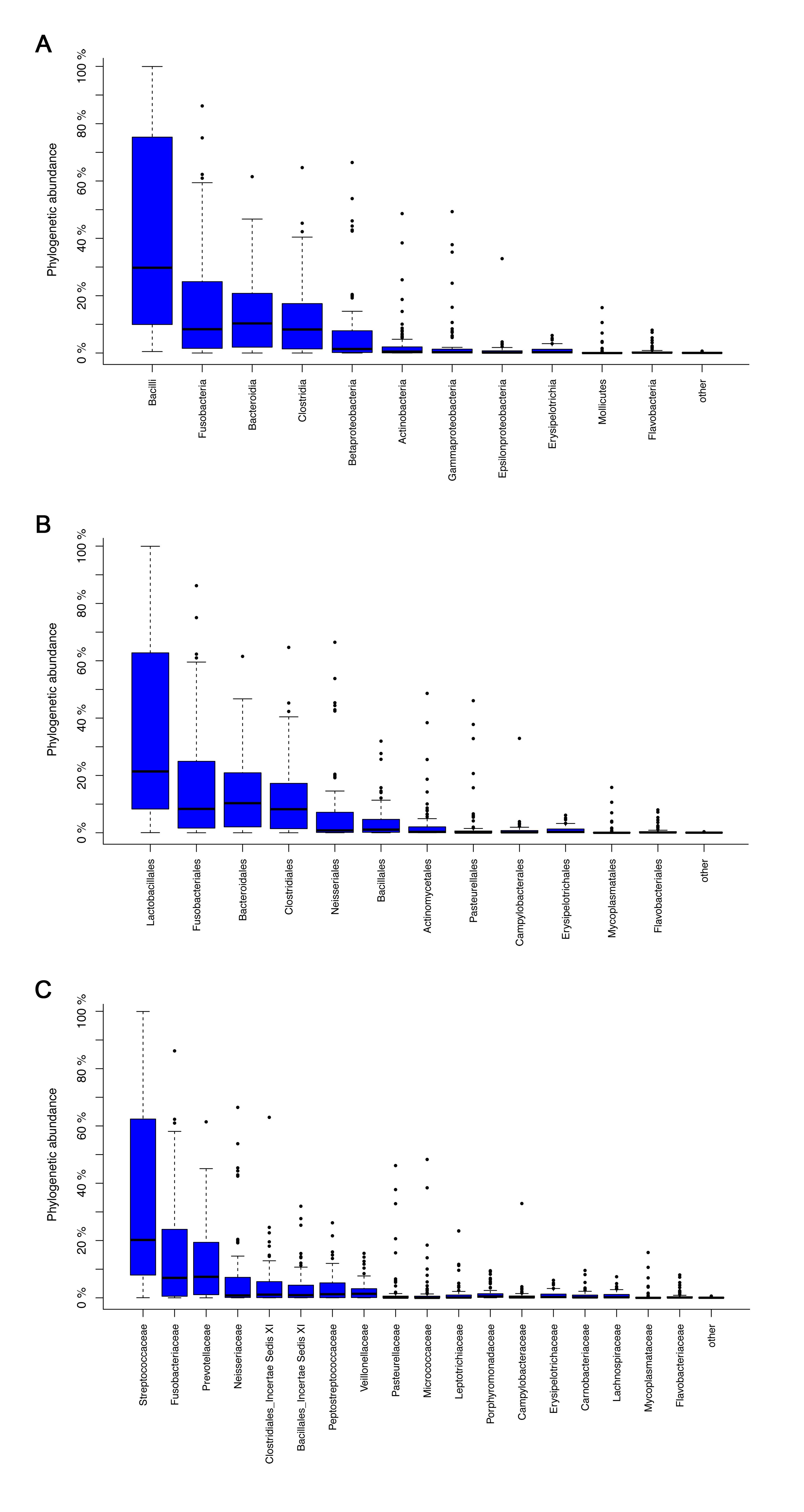

Supplement: Figure S2 — The major abundant communities in the larynx at the level of classes (A), orders (B), families (C). Plotted values are mean sequence abundances in each class, order, and family. (TIF) [file pone.0066476.s002.tif]

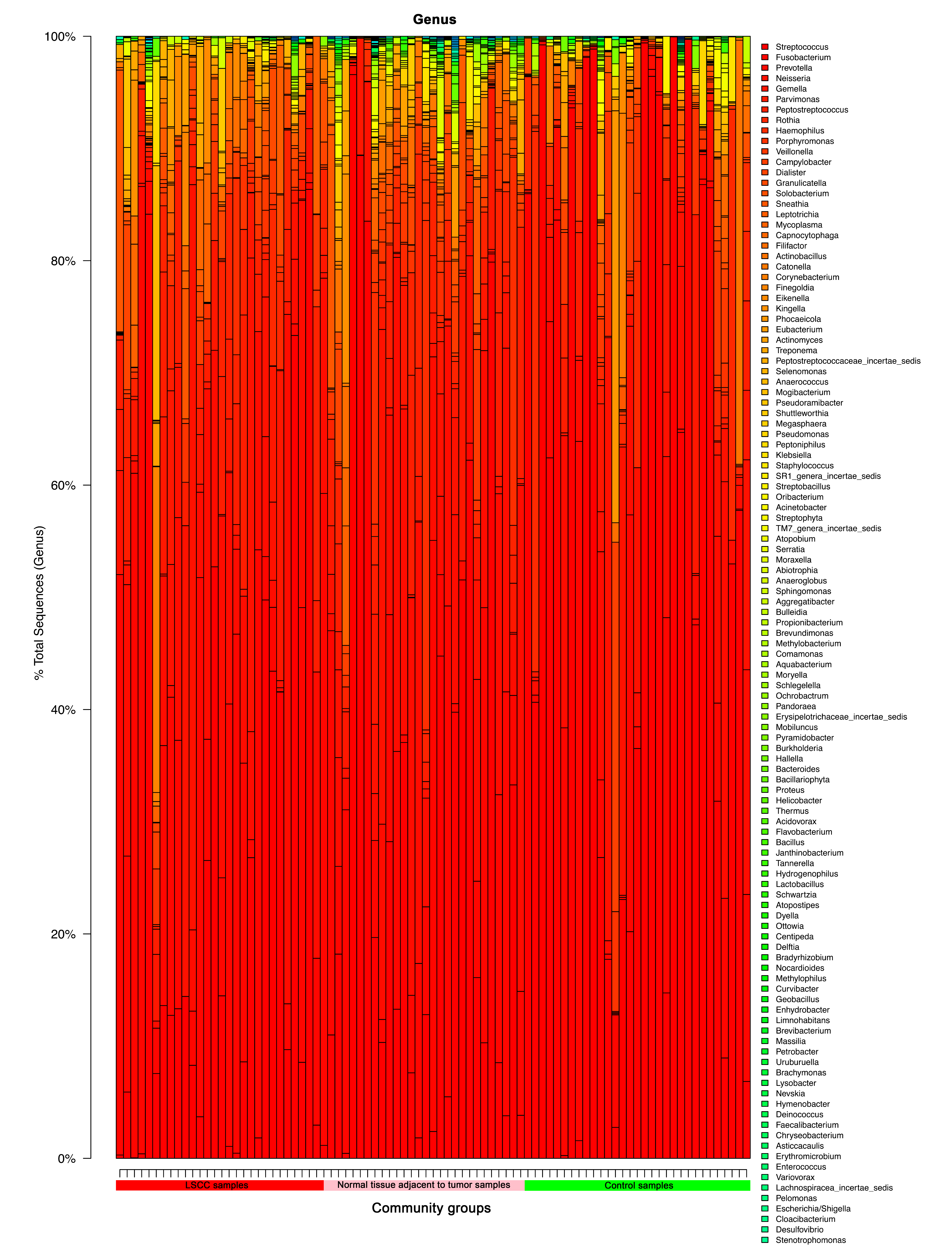

Supplement: Figure S3 — The detailing percentage of the main genera with each laryngeal sample that included LSCC tumor group, normal tissue adjacent to tumor group, and control group. Each color is an individual genus, and each column is a laryngeal tissue sample. Color bars in the right indicate the genera detected in the current study. (TIF) [file pone.0066476.s003.tif]

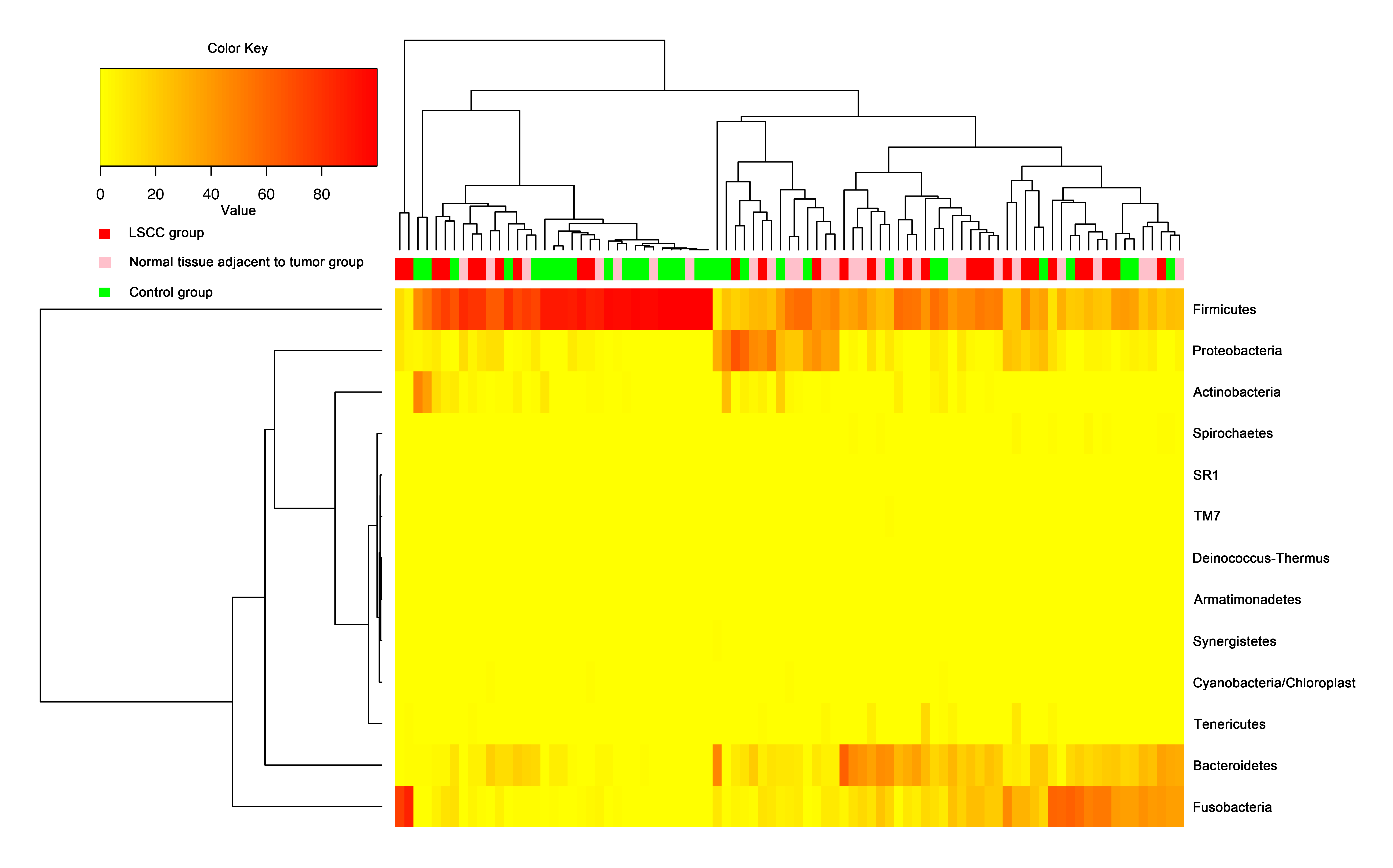

Supplement: Figure S4 — Heatmap of the percentage of the phyla in the larynx of each individual. Complete linkage clustering of samples of the three groups (LSCC tumor, normal tissue adjacent to tumor, and control groups) based on phyla composition and abundance in communities. Each row is an individual phylum, and each column is a laryngeal sample. Color key and color bars are presented in the top-left corner. (TIF) [file pone.0066476.s004.tif]

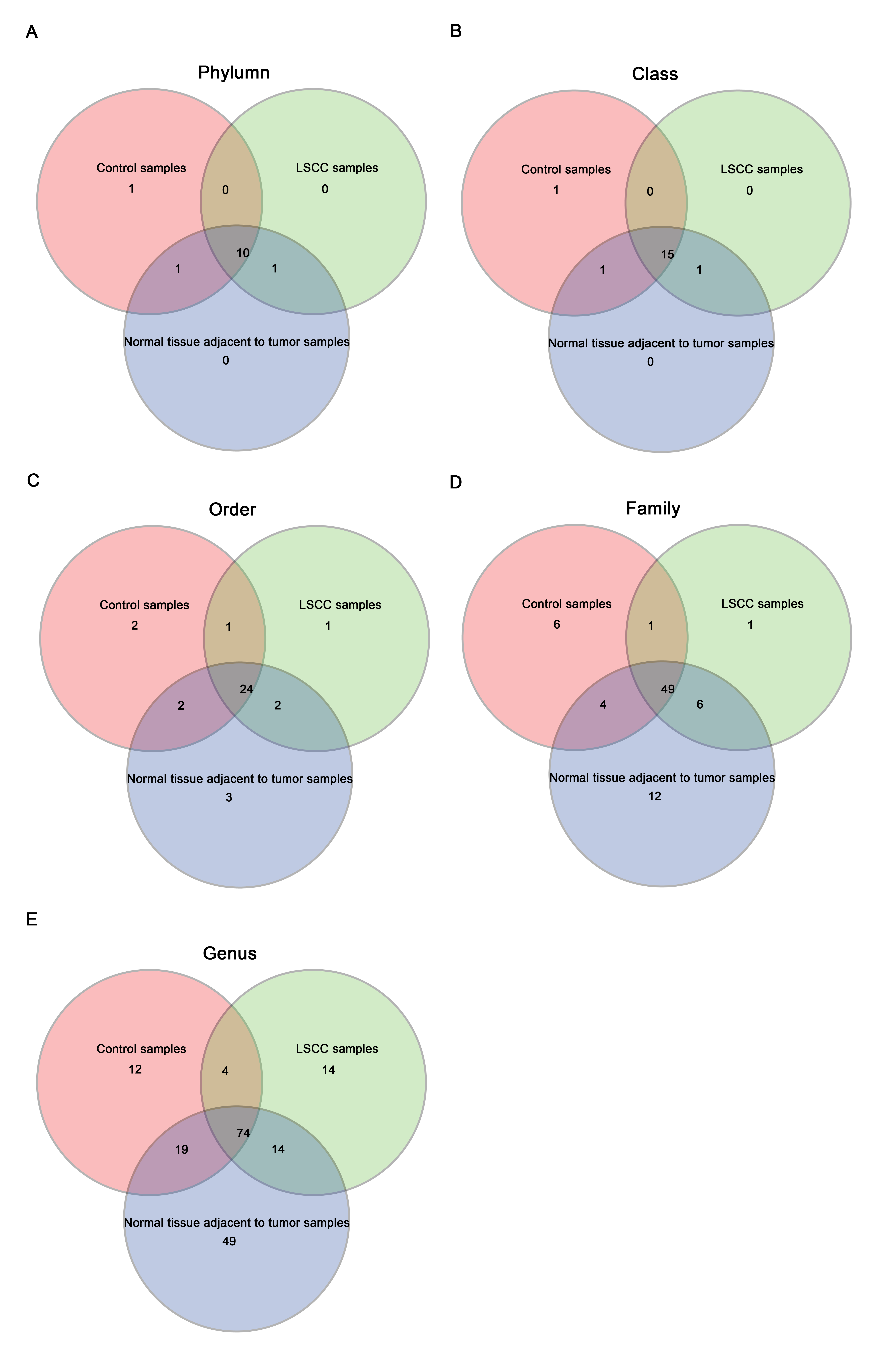

Supplement: Figure S5 — Venn diagrams for overlap among the three groups (LSCC tumor, normal tissue adjacent to tumor, and control groups) at the levels of phyla (A), classes (B), orders (C), families (D), and genera (E). (TIF) [file pone.0066476.s005.tif]

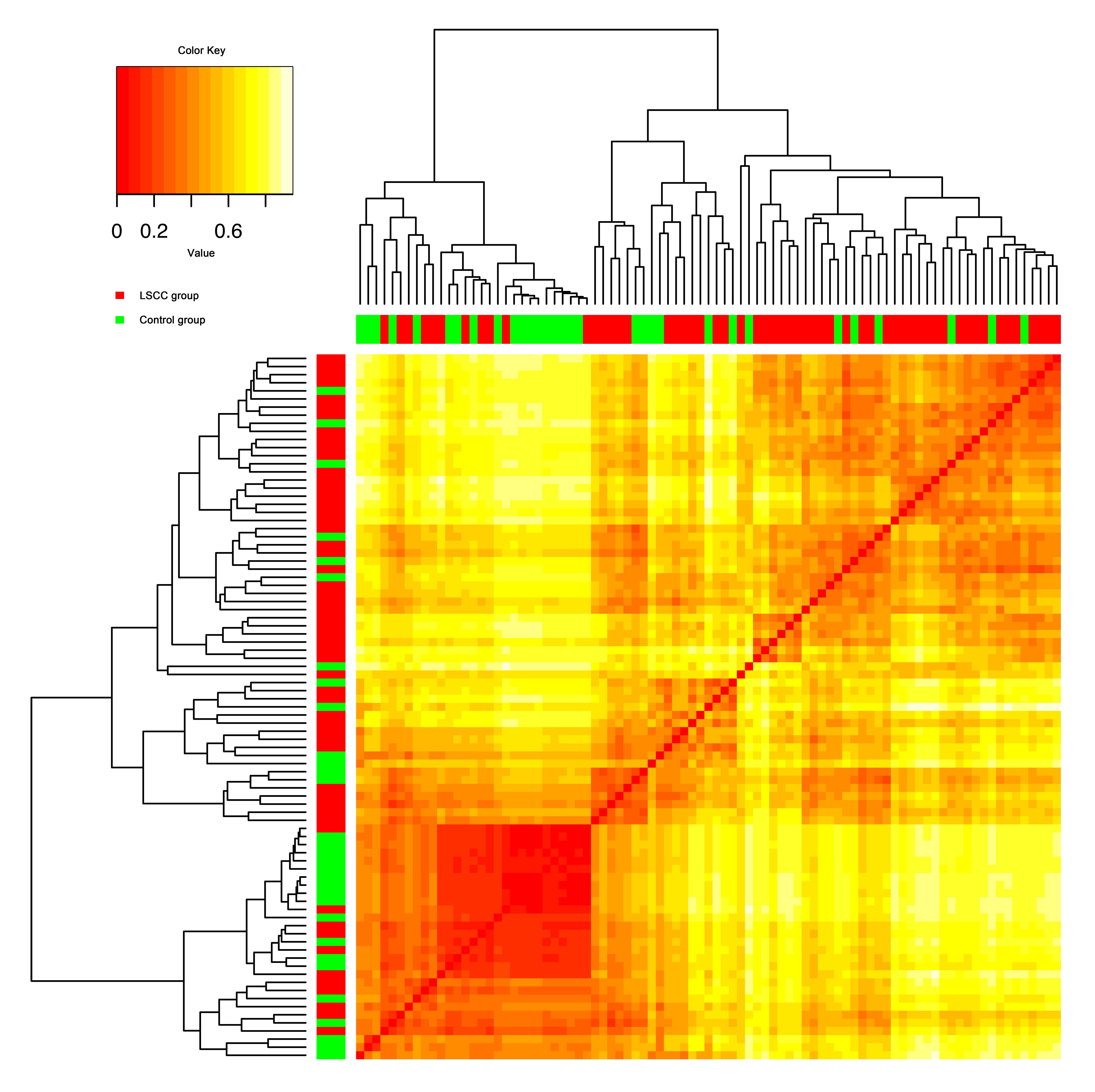

Supplement: Figure S6 — Correlogram of each laryngeal sample. The correlogram was build by community composition and abundance in laryngeal cancer patients and controls. Some samples tended to group together according to cancer group or control status. Color key and color bars are indicated in the top-left corner. (TIF) [file pone.0066476.s006.tif]

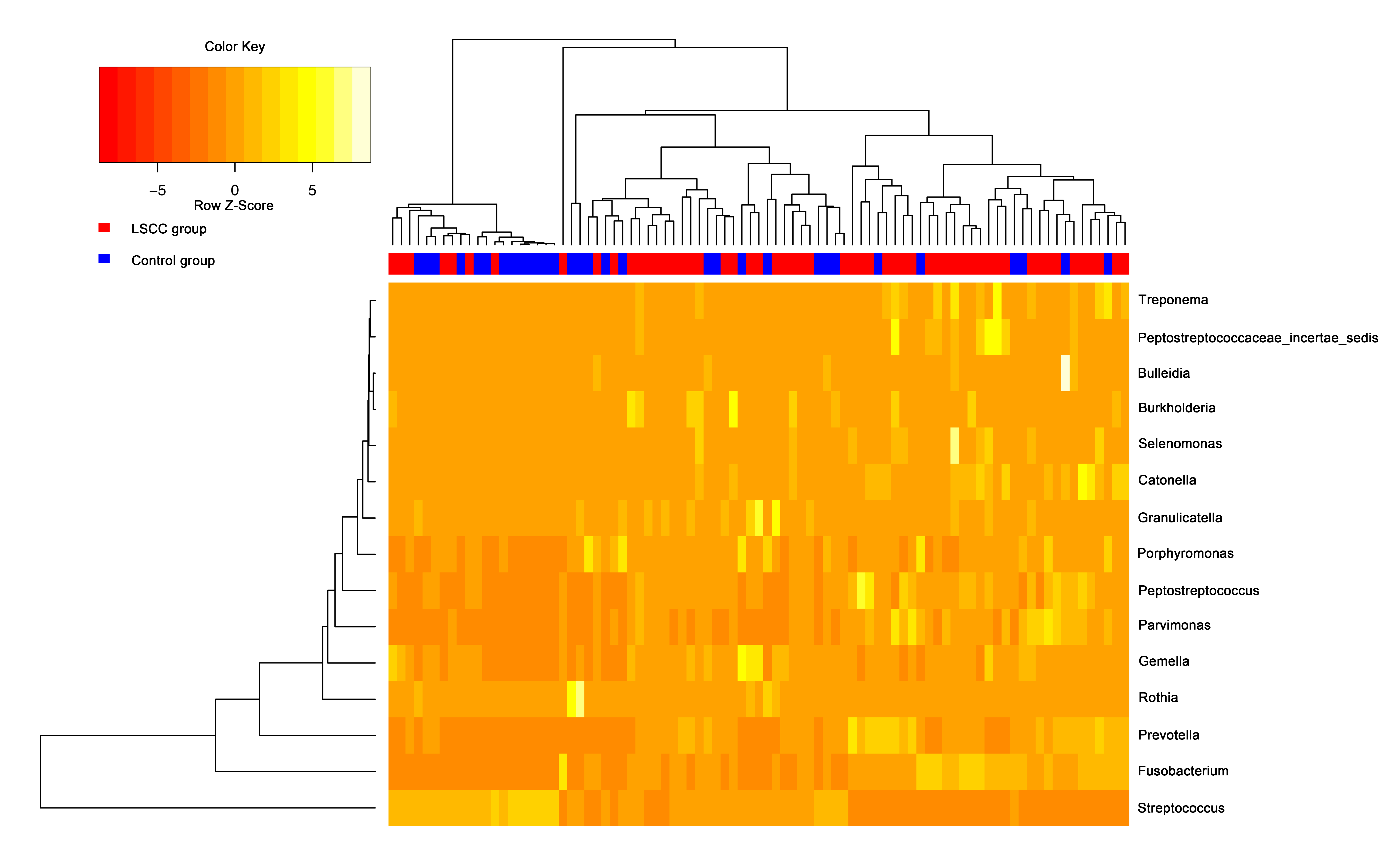

Supplement: Figure S7 — Heatmap of the abundance of the divergent genera in the laryngeal cancer patients and controls. The composition and prevalence of 15 genera were significantly different between laryngeal cancer group and controls investigated by PLS-DA, LOOCV and MANOVA analyses. Color key and color bars are indicated in the top-left corner. (TIF) [file pone.0066476.s007.tif]
