# Supplementary material for: The Composition of Microbiome in Larynx and the Throat Biodiversity between Laryngeal Squamous Cell Carcinoma Patients and Control Population
Source: PLoS One. 2013 Jun 18;8(6):e66476. doi: 10.1371/journal.pone.0066476 (PMC3688906; doi:10.1371/journal.pone.0066476)
Supplement: Table S1 — The community composition difference between laryngeal cancer and control groups. (DOCX) [file pone.0066476.s008.docx]

Table S1. The community composition difference between laryngeal cancer and control groups.

|  | Laryngeal cancer group | Control group | *p* value ^＊^ |
| --- | --- | --- | --- |
|  | % | % |  |
| **Phyla** |  |  |  |
| *Firmicutes* | 47.3 | 64.7 | 0.004 |
| *Fusobacteria* | 20.9 | 8.8 | 0.002 |
| *Bacteroidetes* | 17.0 | 10.4 | 0.048 |
| *Tenericutes* | 1.0 | 0.1 | 0.01 |
| *Spirochaetes* | 0.5 | 0.1 | 0.01 |
| *Synergistetes* | 0.01 | 0.05 | <0.001 |
| **Genera** |  |  |  |
| *Streptococcus* | 25.1 | 56.1 | 0.002 |
| *Fusobacterium* | 19.1 | 7.9 | 0.002 |
| *Prevotella* | 15.0 | 6.9 | 0.01 |
| *Parvimonas* | 4.6 | 1.3 | 0.002 |
| *Peptostreptococcus* | 3.6 | 0.6 | 0.002 |
| *Dialister* | 1.1 | 0.2 | 0.01 |
| *Mobiluncus* | 0 | 0.07 | <0.001 |
| *Erysipelotrichaceae incertae sedis* | 0.04 | 0 | <0.001 |
| *Serratia* | 0.1 | 0.02 | <0.001 |
| *Proteus* | 0.03 | 0 | <0.001 |
| *Pyramidobacter* | 0.01 | 0.05 | <0.001 |
| *Hallella* | 0.01 | 0.05 | <0.001 |
| *Methylobacterium* | 0.1 | 0.01 | 0.002 |
| *Peptostreptococcaceae incertae sedis* | 0.5 | 0.04 | 0.002 |
| *Selenomonas* | 0.5 | 0.04 | 0.004 |
| *Mycoplasma* | 1.0 | 0.1 | 0.01 |
| *Catonella* | 0.7 | 0.2 | 0.01 |
| *Aggregatibacter* | 0.1 | 0.02 | 0.01 |
| *Staphylococcus* | 0.2 | 0.007 | 0.01 |
| *Treponema* | 0.5 | 0.1 | 0.01 |
| *Schlegelella* | 0.05 | 0.01 | 0.01 |
| *Moraxella* | 0.1 | 0 | 0.02 |
| *Capnocytophaga* | 0.8 | 0.2 | 0.02 |
| *Helicobacter* | 0.03 | 0 | 0.02 |
| *Pandoraea* | 0.04 | 0.01 | 0.03 |
| *Abiotrophia* | 0.1 | 0.02 | 0.04 |

^＊^ *p* value were tested from metastats analysis.
